# Quercetin Is An Active Agent in Berries against Neurodegenerative Diseases Progression through Modulation of Nrf2/HO1

**DOI:** 10.3390/nu14235132

**Published:** 2022-12-02

**Authors:** Al Borhan Bayazid, Beong Ou Lim

**Affiliations:** 1 Medicinal Biosciences, Department of Applied Life Science, Konkuk University, Chungju 27478, Republic of Korea; 2 Human Bioscience Corporate R&D Center, Human Bioscience Corp. 268 Chungwondaero, Chungju 27478, Republic of Korea

**Keywords:** quercetin, oxidative stress, cognition, amyloid beta, tau, alpha synuclein

## Abstract

Berries are well-known fruits for their antioxidant effects due to their high content of flavonoids, and quercetin is one of the potent bioactive flavonoids. Although oxidative stress is an inevitable outcome in cells due to energy uptake and metabolism and other factors, excessive oxidative stress is considered a pivotal mediator for the cell death and leads to the progression of neurodegenerative diseases (NDDs). Furthermore, oxidative stress triggers inflammation that leads to neuronal cell loss. Alzheimer’s, Parkinson’s, Huntington’s disease, amyotrophic lateral sclerosis, multiple sclerosis, and so on are the main neurodegenerative diseases. Hence, AD and PD are the most affected NDDs and cause the most lethality without any effective cure. Since AD and PD are the most common NDDs, therefore, in this study, we will describe the effect of oxidative stress on AD and PD. Targeting oxidative stress could be a very effective way to prevent and cure NDDs. Thus, the nuclear factor erythroid 2–related factor 2 (Nrf2) and heme oxygenase-1 (HO1) are potent endogenous antioxidant modulatory pathways, which also show cytoprotective activities. Modulation of Nrf2/HO1 signaling pathways through a biological approach could be an effective way to treat with NDDs. Quercetin is a natural polyphenol, which protects neurodegeneration, remarkably by suppressing oxidative stress and inflammation. Thus, quercetin could be a very effective agent against NDDs. We will discuss the benefits and challenges of quercetin to treat against NDDs, focusing on molecular biology.

## 1. Introduction

Neurodegenerative diseases (NDDs) are a heterogeneous group of diseases, as portrayed by leisurely moderate neuronal cell death [1]. The etiology of neurodegenerative diseases has not yet been completely explained; being that as it may, expanded oxidative stress has been recommended as one of the possible normal etiologies in different NDDs. Excessive oxidative stress might incite cell harm, the impedance of the DNA fixed framework, and mitochondrial brokenness, all of which have been known as key components in the speed increase in the maturing system and the advancement of NDDs [2], such as Alzheimer’s disease (AD) and Parkinson’s disease (PD). AD is the most common NDDs and it affects 10% to 50% of the elderly population, PD is the second most common NDD after AD [3]. Therefore, there have been endeavors to discover specialists that can secure against oxidative stress and possibly treat NDDs [4,5]. We concentrate on the major pathophysiological pathways of oxidative stress to the onset of NDDs, particularly in AD and PD. Furthermore, we will layout the current information on the accessible proof for the anticipation and treatment of NDDs and future headings for the ability of cell reinforcement supplementation with improved adequacy.

Mitochondria are the primary cellular producers of oxygen and contain various redox proteins equipped for moving single electrons to oxygen, producing the reactive oxygen species (ROS) superoxide (O_2_^−^). Mitochondrial chemicals are so far known to create ROS incorporating tricarboxylic acid (TCA) cycle enzymes aconitase (ACO) and α-ketoglutarate dehydrogenase (KGDH); the electron transport chain (ETC) edifices I, II, and III; pyruvate dehydrogenase (PDH) and glycerol-3-phosphate dehydrogenase (GPDH); dihydroorotate dehydrogenase (DHOH); and the cytochrome b5 reductase (B5R) and monoamine oxidases (MAO). Electron exchange with oxygen, producing superoxide, is more likely when these redox transporters carry many electrons and the probable energy of motion is high, as evidenced by the high mitochondrial membrane potential [6]. ROS is reduced when accessible electrons are few and the exchange energy can be low. Mitochondria also contain a broad cell reinforcement protection framework to detoxify the ROS produced by the responses depicted previously [7,8]. Nonenzymatic components of the framework incorporate α-tocopherol, cytochrome C, and GSH. Enzymatic parts incorporate MnSOD (manganese superoxide dismutase), glutathione peroxidase (GP_X_), catalase, phospholipid hydroperoxide glutathione peroxidase, glutathione reductase (GR), peroxiredoxins (PRX3/5), glutaredoxin (GR_X2_), thioredoxin (TRX2), and thioredoxin reductase (TRXR2) [9,10]. The recovery of GSH and diminished TRX2 relies upon nicotinamide adenine dinucleotide phosphate (NADPH), which have been acquired from substrates (through isocitrate dehydrogenase, IDH, or malic catalyst, ME) or the film potential through nicotinamide nucleotide transhydrogenase (NNT). In this way, ROS age and cancer prevention agent safeguards are likewise attached to the redox and vigorous conditions of mitochondria. glutathione disulfide (GSSG), lipid hydroxide (LOH), LOOH, and lipid hydroperoxide; o—oxidized state; r—decreased state. In basically and practically flawless mitochondria, a pivotal cancer prevention agent protection limit adjusts the ROS age. Moreover, there is minimal net ROS creation. Mitochondrial harm with the decline of the cancer prevention agent protection limit is essential for net ROS production. When this happens, an endless loop may follow, whereas ROS could be detrimental to mitochondria. This causes the aggravation of the abnormal aging and misfortune or utilization of the cancer prevention agent limit [11]. The nuclear factor erythroid 2–related factor 2 (Nrf2) is a potent antioxidant mediator protein and usually binds to KEAP-1 (cytosol with Kelch-like ECH-associated protein 1) in the cytoplasm [10,12]. Nrf2 activates through disassociation with the KEAP-1 and translocates into the nucleus [12].

Quercetin is a natural polyphenol, and cumulative shreds of evidence reported its effects on anti-inflammatory, anticancer and antioxidant activities [13,14,15,16]. Quercetin is found abundantly in vegetables and fruits (berries), which target several biomolecules and enzymes [17,18]. Quercetin is a cost-effective polyphenol found in a large number of plants. Quercetin is classified as a flavanol (a type of polyphenol), one of the six subclasses of flavonoid compounds. Flavonoids are a family of plant compounds that share a similar flavone skeleton (a tricyclic molecule with a hydroxyl group [OH] attached). Various other substitutions can occur, creating subclasses of flavonoids and various compounds within those subclasses. Flavonoids also occur as either glycosides (sugar-bound [glycosyl groups]) or aglycones (sugar-bound [non-glycosyl]) [19,20] that may increase bioavailability. Moreover, glycol-conjugated can bind with targeted cell receptors and could be used as a prodrug delivery system. There is increasing evidence that quercetin has therapeutic potential in the prevention and treatment of a variety of diseases, including cardiovascular disease, cancer, and neurodegenerative diseases [18,19,20,21]. Quercetin suppresses inflammatory mediators and ROS via modulating Nrf2/HO1 signaling pathways in neuronal cells [22], as illustrated in the graphical abstract. Mechanistically, quercetin has been shown to exert antioxidant, anti-inflammatory, and cancer-fighting activities in many cell and animal models by regulating the signaling pathways and gene expression involved in these processes.

## 2. Berries as Source of Quercetin

Quercetin is a unique flavanol that has been widely studied by researchers over the past forty years and is abundantly found in plants and fruits. Shikimic acid and glycolytic are the primitive steps for secondary metabolite biosynthesis, and then various enzymatic, extrinsic factors modify the secondary metabolite synthesis such as quercetin [23]. Berries are rich in polyphenols, flavonoids, and anthocyanins and are great sources of quercetin. Whortleberry contains about 158 mg/kg, lingonberry about 145 mg/kg, cranberry about 121 mg/kg, and blueberry about 99.9 mg/kg, respectively [24,25] as mentioned in Table 1. The content of quercetin varies according to the variety of strains, locations, and cultivation. Nevertheless, berries are well-known for their rich content of quercetin and many other polyphenols.

## 3. Metabolism of Quercetin

Quercetin is a flavonoid (a subtype of polyphenol) that absorbs a small quantity in the intestine from its source such as berries, onions, herbs, and so on [26]. The intestinal epithelial cells facilitate the quercetin to the circulatory system through the gastrointestinal (GI) tract. The quercetin metabolism mainly consists of several conjugation reactions catalyzed by sulfotransferases, uridine-5′-diphosphate glucuronosyl transferase, and catechol-O-methyl-transferase, ultimately forming a link. glucuronide metabolism and sulfation or methylation. UGT1A1, UGT1A8, and UGT1A9 appear to be active in the glucuronidation of quercetin [27,28]. Once in the bloodstream, more than 80% of quercetin metabolites are bound to plasma proteins, mainly albumin. Quercetin naturally exists in many different forms. The form of quercetin found in berries is glycone or carbohydrate conjugates, such as (quercetin-3-O-glucoside, quercetin-3-rutinoside, etc), which often act as a pigment that gives color to many fruits and vegetables. In many studies, quercetin’s chemical properties, mechanisms of absorption, metabolism, bioavailability, food sources, bioactivity, and potential health-promoting mechanisms have been exhibited [29,30]. Quercetin is known to be an antioxidant, anti-inflammatory, cardioprotective, and antiobesity compound. It is said to be effective against cardiovascular diseases, cancer, diabetes, neurological diseases, obesity, allergies, asthma, and allergic diseases. Quercetin can bypass the BBB and is found in the brain in vivo in previous studies [31,32].

### 3.1. Effects of Quercetin on Cognitive Impairments

Cognition is a neuropsychological term that refers to a range of mental processes for acquiring learning, experiences, perception, thought, memory, and so on. The growing evidence of the neurobiological bases of synaptic plasticity and memory has opened new avenues for the development of cognitive-enhancing drugs that can be used in the treatment of cognitive impairments. Memory is associated with neuropsychological disorders; the neuroregulatory systems that influence memory formation include stress hormones as well as multiple neurotransmitters and neuropeptide signaling pathways. Here, we review some of the findings on memory enhancement by drugs acting on the neuroregulatory system and discuss possible effects. Cognitive impairments cause lots of neurological and psychological disorders and pivotal symptoms of various NDDs, such as AD, PD, MS, and so on [33]. Cognitive impairment refers to turning down intellectualities, memories, reasoning, and so on [34]. Cognitive impairments lead to dementia. The etiology of cognitive impairments is not fully understood. Previous studies illustrated that cognitive impairments are caused by oxidative stress, neurotoxicity, inflammations, and many more pathological conditions, and quercetin plays a vital role in mitigating cognitive impairments [33,35]. Moreover, it has been proven that berries have an improved cognitive function in preclinical and clinical studies [36]. Quercetin improves spatial learning, neuroplasticity, and overall cognition in aged and dementia-affected mice [37,38,39]. Quercetin could be the potent bioactive in berries to alleviate cognitive impairments. Quercetin ameliorated learning and memory impairments by improving in the Morris water maze (MWM) test [40,41], Y-maze test [42], radial arm maze [43], novel objective recognition (NOR) task [44], passive avoidance test [42], and elevated plus maze test [41] in rodent models.

### 3.2. Effects of Quercetin on Oxidative Stress

Oxidative stress results from ROS, which is produced by many essential physiological processes such as metabolism, respiration, and intrinsic and extrinsic cellular factors. ROS is a byproduct of aerobic metabolism. Quercetin has been reported for remarkably restored oxidative stress by reducing ROS and reactive nitrogen species (RNS) through modulating cellular antioxidant mechanisms. ROS consists of superoxide anions (O_2_-), hydrogen peroxide (H_2_O_2_), hydroxyl radicals (OH·), and so on. They all have unique chemical properties that give them reactivity against different biological targets. ROS is often related to the principle of oxidative stress, and ROS has been thought to cause pathology by damaging lipids, proteins, DNA, and other macromolecules. H_2_O_2_ is made from superoxide produced by mitochondria and NADPH oxidase. Superoxide is formed by the single-electron reduction of molecular oxygen and is rapidly converted intracellularly to H_2_O_2_ by superoxide dismutase and then neutralized by the catalase enzyme. SOD is mainly localized in the cell membrane cleft and mitochondria, while SOD is localized in the mitochondrial matrix. SOD prevents the accumulation of superoxide, which damages and inactivates proteins containing iron–sulfur clusters. ROS are neutralized by the cellular antioxidant enzymes that are produced by the cellular antioxidant mechanisms. Apoptosis may take place when the ROS level is imbalanced by exceeding cellular antioxidant activity [45]. Antioxidant mediators such as the Nrf2/HO1 pathway are important in protecting against neurodegeneration. An excess of ROS induces the production of inflammatory factors that prolong neuronal cell death and exacerbates mitochondrial function, leading to apoptosis. Several studies have reported that the central cause of NDD is oxidative stress-mediated cell death by disrupting cellular antioxidant pathways [45,46,47,48,49,50]. Oxidative stress triggers a signaling cascade that causes mitochondrial dysfunction, which leads to neuronal cells loss. In addition, oxidation stress can cause chronic inflammation and maintain the death of inflammatory cells. Quercetin mitigates oxidative stress-mediated inflammation in microglia and other neuronal cells [51,52]. The neurons are protected by quercetin by adjusting the Nrf2 and HO1 antioxidant signal path in vivo and in vitro. Quercetin has placed the route-signaling Nrf2 and HO1 and has specified expression of antioxidant enzymes, such as SOD and GPX, etc., in the brain [53,54], which is consistent with the quercetin-initiated downregulation of inflammatory cytokines and apoptotic markers by positively modulating the Nrf2/HO1 cellular antioxidant defense mechanism [4]. Drug synergisms are clinically significant when lower-dose combinations produce greater efficacy with fewer side effects than individual doses of each drug. Moreover, quercetin showed synergistic effects with kaempferol and/or pterostilbene and increased the bioavailability [55], and it also remarkably attenuated oxidative stress through the Nrf2/ARE pathway.

### 3.3. Effects of Quercetin on P-53-Mediated Apoptosis

Quercetin is considered a suppressor of hyperactive P-53, which is triggered by several intrinsic and extrinsic factors that lead to cell death [56,57,58], and meanwhile, it also stabilizes P-53 in cancer cells [59]. Neurodegeneration is caused by the upregulation of P-53, which was discovered 40 years ago and has been widely studied and causes AD, PD, HD, and so on. P-53 can be triggered by various cellular abnormalities such as damaged DNA, ROS, MAPK, and so on [60,61], but sometimes without extended stress, P-53 hyperactivation takes place by ROS, which are byproducts of normal respiration and metabolism [62]. Potent transcription factor P-53 has many cellular functional activities: it may induce cell cycle arrest, DNA damage, and so on [63]. P-53 is an upstream apoptotic mediator, and quercetin significantly ameliorates P-53 and its downstream apoptotic mediators against several stimuli [63,64,65]. The mitochondria are the main energy-producing systems in the cell and regulate key factors of cell death such as P-53, BAX, cytochrome C, and caspases [2,66,67]. Apoptosis is a process of regular programmed cell death. Mitochondrial dysfunction is thought to be a possible key to the deregulation of the apoptotic pathway [67,68]. The high P-53 level is a marker of neurodegeneration. Indeed, this has been confirmed, for example, in the case of proteolytic products of the precursor protein Aβ, which is a transcriptional regulator of the P-53 gene and has been shown to act as a transcriptional regulator. Oxidative stress is one of the main causes of mitochondrial dysfunction, leading to abnormal apoptosis. Furthermore, mitochondrial dysfunction can also lead to the onset/exaggeration of neurodegenerative diseases (NDDs) by affecting apoptotic pathways, by influencing chronic inflammation, or by apoptosis. The expression of C9orf72(PR)50 in P-53 stable neurons activates genome-wide regulatory factors that are enriched for P-53 binding sites and leads to gene upregulation. As a major P-53 target, we then sought to directly test whether P-53 is required for C9orf72(PR)50-induced neurodegeneration and proteolytic separation. P-53 KO neurons were able to completely counteract the toxicity of C9orf72(PR)50 accumulation [69]. Furthermore, the impaired mitochondrial function also disrupts autophagic clearance (i.e., mitophagy) and induces aggregation of filamentous or misfolding proteins, e.g., amyloid beta (Aβ) and α-synuclein, leading to Alzheimer’s disease and Parkinson’s disease [70]. However, excess of cytochrome c, a component of ETC release due to oxidative stress and/or increased P-53 secretion, is an indication of damaged mitochondrial membrane potential [2]. Quercetin has demonstrated neuroprotection by markedly restoring P-53 and downstream apoptotic markers such as BAX, Cyto C, and caspase cascades in neurons [56,64]. Furthermore, quercetin repairs the P-53 triggering factors. Thus, the downregulation of apoptotic biomarkers in brain tissue could be a significant effect of neuroprotection.

### 3.4. Effects of Quercetin on Neuroinflammation

Quercetin reduces inflammation in the nervous system by downregulating proinflammatory cytokines (TNF, ILs, IFN, etc.) and mediators. Inflammation is an inevitable immune response towards antigens, pathogens, damaged cells, and so on [70]. Precisely, an adverse effect of immune response is called inflammation, and the cells require homeostasis other than the hyperactivation of inflammatory markers. Neuroinflammation is considered the cause of several psychiatric disorders, including anxiety, depression, schizophrenia, AD, and CNS epilepsy. The immune system is more susceptible to age. Long-term, severe post-infection sepsis can lead to depression and anxiety in older patients. Various epidemiological studies have reported that neuroinflammation is an important risk factor and that systemic inflammation is associated with the cause of neuropsychiatric disorders. Recently, targeted treatments for neuroinflammation have been proposed as new therapeutic tools for the control of neuropsychiatric disorders. Inflammation and oxidative stress are interrelated and show detrimental effects on cell survival. For instance, TLR4, LPS activates NADPH oxidase, which is abundantly expressed in macrophages and induces ROS production. This increases macrophage activation and ultimately causes excessive inflammation [71], which leads to inflammatory neuronal cell death. Inflammation is an adverse immune response to pathogens, antigens, and so on, and inflammation in neurons is called neuroinflammation. Neuroinflammation is a pivotal factor for neurodegeneration that triggers AD, PD, and other NDDs. Previous studies have shown that LPS administration significantly increases the expression levels of inflammatory cytokines and mediators in the hippocampus. The expression of TNF-α and IL-1β mRNA is dynamically regulated by immune cells in the hippocampal inflammatory response [72,73]. However, quercetin has shown the ability to mitigate the abnormalities such as anxious behavior and neuroinflammation by consistently lowering LPS-induced IL-6 and IL-1β levels. Furthermore, these results also suggested that the inflammatory response to LPS infiltration significantly increased COX-2 levels by regulating the MAPK/NF-κB signaling pathway in the hippocampus. Previous studies have also shown that NF-κB, which mainly regulates the inflammatory response, is activated by LPS and inflammatory cytokines infiltration. Quercetin addresses LPS and other inflammatory mediator-induced behavioral disorders such as anxiety by significantly inhibiting COX-2 levels through NF-κB regulation [11,74]. Quercetin alleviates inflammatory markers, attenuates microgliosis and astrogliosis [75], and helps to maintain homeostasis as a natural antioxidant and anti-inflammatory agent. Moreover, quercetin promotes autophagic clearance, maintains the neurons in homeostatic conditions, and mitigates inflammatory and oxidative stress-mediated neurodegeneration [76]. NLRP3 inflammasome activation is strongly associated with the pathogenesis of NDDS, and quercetin has been shown to remarkably restore inflammasome activation and protect NDDs progression [77].

### 3.5. Effects of Quercetin on Amyloid-β and Tau

**Figure 1:**
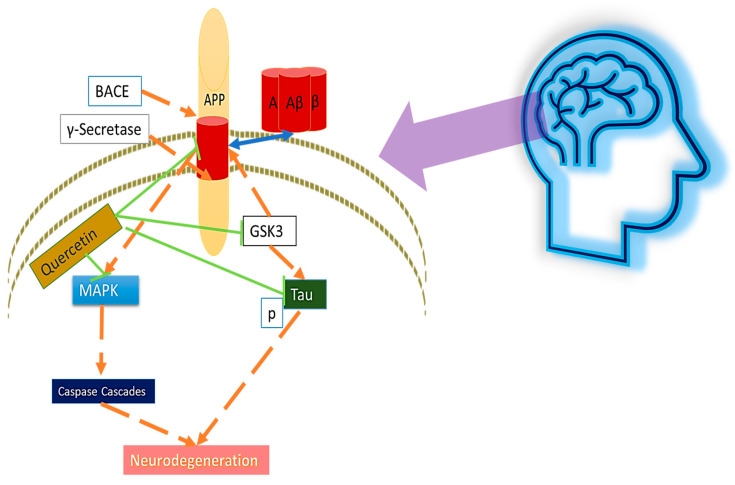
The effects of quercetin on amyloid beta and tauopathy of neurodegeneration.

Aβ aggregation and tau hyperphosphorylation play a central role in the progression of AD, which is the most common NDD and causes millions of deaths [78,79,80]. The tau protein, and its ability for microtubule formation, had been discovered in 1975. Mammalian cell culture systems, both clones, and primary cells have been shown to be invaluable in elucidating the signaling pathways that regulate tau phosphorylation and the impact of phosphorylation events specific to tau function. GSK-3β is an attention-grabbing protein kinase just as tau kinase. Early studies suggest that co-transfection of tau and its GSK-3β into non-neuronal cells strongly increases tau phosphorylation at multiple epitopes. Furthermore, it has been demonstrated that co-expression of GSK-3β and tau markedly altered the binding of tau to microtubules [81], and quercetin restored tau protein dysregulation and helped to gain functionalities [82,83]. Despite the many hypotheses that explain the pathology of AD, the Aβ hypothesis is the main theory. Previous studies have confirmed that overproduction and accumulation of Aβ in the brain cause subsequent pathological events such as neuroinflammation, hyperphosphorylation of tau, and loss of neuronal cells. Aβ produces from the apolipoprotein (APP) by cleaving the enzymes called BACE and γ-secretase, and also the loss of cellular integrity and cognitive functions, and so on [84,85,86]. Many studies suggest that investor relations in the brain may play an important role in early AD. Studies have reported a close relationship between insulin resistance (IR) and Aβ accumulation, where Aβ aggregation causes neuroinflammatory and c-Jun N-terminal kinase (JNK) activation of the serine residue insulin receptor substrate 1 (IRS-1) [72]. It has been suggested that it can cause tau phosphorylation. Ultimately, it leads to a pathological cascade such as tau dysfunction and neuronal loss [87]. Various studies reported that polyphenols may alleviate Aβ caused toxicity through modulation of antioxidant activities [88], and that quercetin also elucidated primary neuroprotective effects in Aβ induced by suppressing the oxidative stress [89]. An important phenomenon in AD—an increase in the number of dead neurons in the hippocampus—has been shown in an Aβ-injected animal model. Interestingly, quercetin can protect neurons from death and has shown an increase in intracellular tau aggregation due to Aβ. The same results showed in our study that taurine oxidation was increased in the hippocampus of Aβ-induced mice. Important markers of both Aβ levels and AD, including hippocampal tau phosphorylation, were reduced during quercetin treatment. In addition, GSK3β is one of the important kinases involved in tau protein phosphorylation and was involved in the progression of AD pathology [7,87,90], as illustrated in Figure 1. The Aβ 42-induced reduction in GSK-3β phosphorylation was reversed by quercetin treatment, and the hyperphosphorylation of tau Ser404 was reduced. Quercetin has been shown to protect neuronal cell death via modulating BDNF/TrkB pathways [91]. BDNF has been reported help to produce Aβ-specific T-cells [70]. Moreover, quercetin reduces insulin resistance [92]. Nrf2 pathways modulation might be an effective approach to reduce Aβ and tau hyperactivation [93]. Several studies suggest quercetin as a safe and effective agent to prevent age-related progressive neurodegenerative diseases such as AD.

### 3.6. Effects of Quercetin on Apolipoprotein E ε4

Apolipoprotein E synthesizes by the regulatory APOE gene mechanisms, and it is highly associated with the late onset of AD [94,95]. Oxidative stress destabilizes APOE ε4 and causes AD [96], whereas quercetin shows great antioxidant activities. Among the three alleles of APOE, APOE ε4 allele is more relatable to AD, dementia, memory loss, and so on compared to the APOE ε4 suppressed or knock-out model [97,98,99]. The APOE ε4 mechanism related to NDDs is yet to be understood; hence, it has been reported that APOE ε4 affects Aβ aggregations, tauopathy, Lewy bodies, inflammations, and so on, and it has been considered as the main genetic risk factor of AD [99,100,101]. Aβ aggregations are increased by APOE ε4 due to decreases in Aβ clearance that perpetuate AD [102]. Moreover, exogenous Aβ could develop AD by triggering a receptor expressed on myeloid cells 2 (TREM2) and forming senile plaques [103,104]. Single nucleotide polymorphism (SNP) of APOE ε4 phosphorylates tau protein and forms NFT and the loss of neuronal function in CNS, as well as causing neurodegeneration due to the tauopathy of AD [103,105]. Quercetin could reduce AD progression in mice by balancing APOE ε4 [20,97]. APOE ε4 causes mitochondrial dysfunction while Nrf2 protects mitochondria, and increased APOE ε4 decreased the endogenous antioxidants mechanisms [106,107]. Thereby, quercetin might play a tremendous role in AD by normalizing APOE ε4. Quercetin has been shown to reduce the accumulation of Aβ by stabilizing APOE in AD mice [20].

### 3.7. Effects of Quercetin on α-Synuclein

Alpha-synuclein is a presynaptic neural protein that is genetically and neuropathologically associated with PD. Parkinson’s disease is an outcome of the loss of diamagnetic neurons and the second most common NDD after AD [108]. α-synuclein can contribute to the pathogenesis of Parkinson’s disease in a variety of ways, but its aberrant soluble oligomeric conformation, known as protofibrils, disrupts cell homeostasis and involves a variety of intracellular targets, including synapses. Moreover, it is highly associated with oxidative stress, and quercetin has shown remarkable effects [108]. It is widely believed to be a toxic species that causes neuronal cell death in the mediation function. In addition, secreted α-synuclein can have detrimental effects on adjacent cells, including disseminated aggregation, and can contribute to the spread of the disease. It is not clear to what extent alpha-synuclein is involved in all cases of PD, but targeting the toxic function of this protein is new in dysregulation, not only in PD but also in other neurodegenerative diseases. It can lead to the diverse groups of NDDs and modify the treatment strategies, which are called synucleinopathies [109]. A plethora of studies suggest that abnormalities in mitochondria and mitophagy lead to PD. Quercetin, one of the most abundant polyphenolic flavonoids, has many beneficial biological effects on many diseases. Many studies demonstrated the neuroprotective effect of quercetin in vivo and 6-hydroxydopamine (6OHDA)-treated PC-12 cells in a 6OHDA-treated rat model of PD. In vitro, quercetin treatment improves mitochondrial quality control, reduces oxidative stress, raises levels of the mitophagy markers PINK1 and Parkin, and reduces expression of the α-synuclein protein in 6OHDA-treated PC-12 cells. Furthermore, in vivo results show that quercetin administration improves 6OHDA-induced progressive PD-like motor behavior in PD rats, reduces neuronal cell death, and reduces mitochondrial damage and α-synuclein accumulation. Furthermore, it has been demonstrated that the neuroprotective effect of quercetin was suppressed by a knockdown of Pink1 or Parkin [110]. Sirtuins1 and GCN5 are negatively related to α-synuclein, and quercetin modulates Sirtuins1 and GCN5 and alleviates PD [111,112]. Quercetin has exerted many potential effects against PD and ensures promising outcomes as a therapeutic agent in PD patients.

## 4. Clinical Trial of Quercetin

Quercetin has proved to be a very effective agent against NDDs in preclinical studies, indicating a promising outcome in human interventions [113]. Quercetin showed the ability to bypass the BBB and alleviate cognitive impairment, as well as demonstrating neuroimaging markers in aged AD populations [113]. Another study elucidated that 24 weeks of quercetin supplements (50 mg equivalent) prevented cognitive decline by improving depressive symptoms and elevating motivation in an aged double-blinded placebo trial [114]. Quercetin exhibits several molecular and physiological effects on various organisms, including humans. Although quercetin has antioxidant activity, its ability to bind to proteins and regulate their activity suggests that this phytochemical has multiple modes of action against NDDs.

## 5. Limitations and Challenges of Quercetin for Neuroprotection

i. The bootability of quercetin is poor, and in human plasma, free quercetin has not been found after oral ingestion of quercetin [115,116].
ii. Drug interactions with colchicine and alprazolam [117].
iii. A large quantity is required for greater effectiveness [118].

## 6. Conclusions

Oxidative stress and mitochondrial dysfunction are the main causes of neurodegeneration. The increase in neurodegenerative diseases is always horrifying because there is no effective cure. Studies over the past few years have established mechanisms by which quercetin improves brain health in many ways, including improving cognitive abilities. Quercetin will undoubtedly increase as a cultivable field in both scientific research and pharmacological and clinical applications. The lipid nanoparticle of quercetin increased the bioavailability of quercetin and/or glycol-conjugate quercetin could be used as prodrug delivery system and shows more effectiveness against neurodegeneration. Altogether, we envisaged that quercetin could be an effective therapeutic agent against AD and PD along with other NDDs.

## Figures and Tables

**Table 1:** The names of berries and their quercetin content.

Berry	Content (mg/kg)	Ref.
Whortleberry	158	[22,23,24]
Lingonberry	146	
Cranberry	121	
Blueberry	99.9	
Chokeberry	89	
Rowanberry	63	
Sea buckthorn berry	62	
Crowberry	56	
Elderberry juice	6.13	

## Data Availability

Not applicable.

## References

[1] Pedersen J.T., Chen S.W., Borg C.B., Ness S., Bahl J.M., Heegaard N.H., Teilum K. (2016). Amyloid-β and α-synuclein decrease the level of metal-catalyzed reactive oxygen species by radical scavenging and redox silencing. J. Am. Chem. Soc..

[2] Chan D.C. (2006). Mitochondria: Dynamic Organelles in Disease, Aging, and Development. Cell.

[3] Zhang Y.-W., Thompson R., Zhang H., Xu H. (2011). APP processing in Alzheimer’s disease. Mol. Brain.

[4] Luo J.-F., Shen X.Y., Lio C.K., Dai Y., Cheng C.S., Liu J.X., Zhou H. (2018). Activation of Nrf2/HO-1 Pathway by Nardochinoid C Inhibits Inflammation and Oxidative Stress in Lipopolysaccharide-Stimulated Macrophages. Front. Pharmacol..

[5] Arnold S., Kadenbach B. (2012). Cytochrome c Oxidase and Its Role in Neurodegeneration and Neuroprotection. Mitochondrial Oxidative Phosphorylation: Nuclear-Encoded Genes, Enzyme Regulation, and Pathophysiology.

[6] Forman H.J., Zhang H. (2021). Targeting oxidative stress in disease: Promise and limitations of antioxidant therapy. Nat. Rev. Drug Discov..

[7] Moosecker S., Gomes P., Dioli C., Yu S., Sotiropoulos I., Almeida O.F. (2019). Activated PPARγ abrogates misprocessing of amyloid precursor protein, Tau missorting and synaptotoxicity. Front. Cell. Neurosci..

[8] Koh E.-J., Kim K.-J., Choi J., Kang D.-H., Lee B.-Y. (2018). Spirulina maxima extract prevents cell death through BDNF activation against amyloid beta 1-42 (Aβ 1-42) induced neurotoxicity in PC12 cells. Neurosci. Lett..

[9] Bayazid A.B., Jang Y.A., Kim Y.M., Kim J.G., Lim B.O. (2021). Neuroprotective Effects of Sodium Butyrate through Suppressing Neuroinflammation and Modulating Antioxidant Enzymes. Neurochem. Res..

[10] Tonelli C., Chio I.I.C., Tuveson D.A. (2018). Transcriptional Regulation by Nrf2. Antioxid. Redox Signal..

[11] Jabri M.-A., Rtibi K., Sebai H. (2020). Chamomile decoction mitigates high fat diet-induced anxiety-like behavior, neuroinflammation and cerebral ROS overload. Nutr. Neurosci..

[12] Brandes M.S., Gray N.E. (2020). NRF2 as a Therapeutic Target in Neurodegenerative Diseases. ASN Neuro.

[13] Watson R.R. (2011). Complementary and Alternative Therapies and the Aging Population: An Evidence-based Approach.

[14] Bayazid A.B., Jang Y.A. (2021). The Role of Andrographolide on Skin Inflammations and Modulation of Skin Barrier Functions in Human Keratinocyte. Biotechnol. Bioprocess Eng..

[15] Bayazid A.B., Jang Y.A., Jeong S.A., Lim B.O. (2022). Cypress tree (*Chamaecyparis obtusa*) Bark extract inhibits melanogenesis through repressing CREB and MITF signalling pathways in *α*-MSH-stimulated B16F10 cells. Food Agric. Immunol..

[16] Valle I.F.D., Roweth H.G., Malloy M.W., Moco S., Barron D., Battinelli E., Loscalzo J., Barabási A.-L. (2021). Network medicine framework shows that proximity of polyphenol targets and disease proteins predicts therapeutic effects of polyphenols. Nat. Food.

[17] Redford K.E., Abbott G.W. (2020). The ubiquitous flavonoid quercetin is an atypical KCNQ potassium channel activator. Commun. Biol..

[18] Dabeek W.M., Marra M.V. (2019). Dietary Quercetin and Kaempferol: Bioavailability and Potential Cardiovascular-Related Bioactivity in Humans. Nutrients.

[19] García-Viñuales S., Ahmed R., Sciacca M.F.M., Lanza V., Giuffrida M.L., Zimbone S., Romanucci V., Zarrelli A., Bongiorno C., Spinella N. (2020). Trehalose Conjugates of Silybin as Prodrugs for Targeting Toxic Aβ Aggregates. ACS Chem. Neurosci..

[20] Zhang X., Hu J., Zhong L., Wang N., Yang L., Liu C.-C., Li H., Wang X., Zhou Y., Zhang Y. (2016). Quercetin stabilizes apolipoprotein E and reduces brain Aβ levels in amyloid model mice. Neuropharmacology.

[21] Rauf A., Imran M., Khan I.A., Ur-Rehman M., Gilani S.A., Mehmood Z., Mubarak M.S. (2018). Anticancer potential of quercetin: A comprehensive review. Phytotherapy Res..

[22] Lesjak M., Beara I., Simin N., Pintać D., Majkić T., Bekvalac K., Orčić D., Mimica-Dukić N. (2018). Antioxidant and anti-inflammatory activities of quercetin and its derivatives. J. Funct. Foods.

[23] Singh P., Arif Y., Bajguz A., Hayat S. (2021). The role of quercetin in plants. Plant Physiol. Biochem..

[24] Sellappan S., Akoh C.C., Krewer G. (2002). Phenolic Compounds and Antioxidant Capacity of Georgia-Grown Blueberries and Blackberries. J. Agric. Food Chem..

[25] Häkkinen S.H., Kärenlampi S.O., Heinonen I.M., Mykkänen H.M., Törrönen A.R. (1999). Content of the Flavonols Quercetin, Myricetin, and Kaempferol in 25 Edible Berries. J. Agric. Food Chem..

[26] Russo G.L., Russo M., Spagnuolo C. (2014). The pleiotropic flavonoid quercetin: From its metabolism to the inhibition of protein kinases in chronic lymphocytic leukemia. Food Funct..

[27] Docampo M., Olubu A., Wang X., Pasinetti G., Dixon R.A. (2017). Glucuronidated Flavonoids in Neurological Protection: Structural Analysis and Approaches for Chemical and Biological Synthesis. J. Agric. Food Chem..

[28] Lines T.C. (2013). Quercetin-Containing Compositions. US Patents.

[29] Ulusoy H.G., Sanlier N. (2019). A minireview of quercetin: From its metabolism to possible mechanisms of its biological activities. Crit. Rev. Food Sci. Nutr..

[30] Lakhanpal P., Rai D.K. (2007). Quercetin: A versatile flavonoid. Internet J. Med. Update.

[31] Terao J., Murota K., Kawai Y. (2011). Conjugated quercetin glucuronides as bioactive metabolites and precursors of aglycone in vivo. Food Funct..

[32] Xiao J., Kai G. (2012). A Review of Dietary Polyphenol-Plasma Protein Interactions: Characterization, Influence on the Bioactivity, and Structure-Affinity Relationship. Crit. Rev. Food Sci. Nutr..

[33] Bakoyiannis I., Daskalopoulou A., Pergialiotis V., Perrea D. (2019). Phytochemicals and cognitive health: Are flavonoids doing the trick?. Biomed. Pharmacother..

[34] Robertson D.A., Savva G.M., Kenny R.A. (2013). Frailty and cognitive impairment—A review of the evidence and causal mechanisms. Ageing Res. Rev..

[35] Broman-Fulks J.J., Canu W.H., Trout K.L., Nieman D.C. (2012). The effects of quercetin supplementation on cognitive functioning in a community sample: A randomized, placebo-controlled trial. Ther. Adv. Psychopharmacol..

[36] Subash S., Essa M.M., Al-Adawi S., Memon M.A., Manivasagam T., Akbar M. (2014). Neuroprotective effects of berry fruits on neurodegenerative diseases. Neural Regener. Res..

[37] Wang D.-M., Li S.-Q., Wu W.-L., Zhu X.-Y., Wang Y., Yuan H.-Y. (2014). Effects of Long-Term Treatment with Quercetin on Cognition and Mitochondrial Function in a Mouse Model of Alzheimer’s Disease. Neurochem. Res..

[38] Yang S., Wang G., Ma Z.F., Qin L.-Q., Zhai Y.-J., Yu Z.-L., Xue M., Zhang Y.-H., Wan Z. (2019). DietaryAdvancedGlycationEnd Products-InducedCognitive Impairment in Aged ICR Mice: Protective Role of Quercetin. Mol. Nutr. Food Res..

[39] Zingales V., Sirerol-Piquer M.S., Fernández-Franzón M., Ruiz M.-J. (2021). Role of quercetin on sterigmatocystin-induced oxidative stress-mediated toxicity. Food Chem. Toxicol..

[40] Halder S., Kar R., Galav V., Mehta A.K., Bhattacharya S.K., Mediratta P.K., Banerjee B.D. (2015). Cadmium exposure during lactation causes learning and memory-impairment in F1 generation mice: Amelioration by quercetin. Drug Chem. Toxicol..

[41] Priprem A., Watanatorn J., Sutthiparinyanont S., Phachonpai W., Muchimapura S. (2008). Anxiety and cognitive effects of quercetin liposomes in rats. Nanomed. Nanotechnol. Biol. Med..

[42] Choi G.N., Kim J.H., Kwak J.H., Jeong C.-H., Jeong H.R., Lee U., Heo H.J. (2011). Effect of quercetin on learning and memory performance in ICR mice under neurotoxic trimethyltin exposure. Food Chem..

[43] Pu F., Mishima K., Irie K., Motohashi K., Tanaka Y., Orito K., Egawa T., Kitamura Y., Egashira N., Iwasaki K. (2007). Neuroprotective Effects of Quercetin and Rutin on Spatial Memory Impairment in an 8-Arm Radial Maze Task and Neuronal Death Induced by Repeated Cerebral Ischemia in Rats. J. Pharmacol. Sci..

[44] Mert D.G., Turgut N.H., Arslanbas E., Gungor H., Kara H. (2018). The influence of quercetin on recognition memory and brain oxidative damage in a ketamine model of schizophrenia. Psychiatry Clin. Psychopharmacol..

[45] Cavaliere G., Trinchese G., Penna E., Cimmino F., Pirozzi C., Lama A., Annunziata C., Catapano A., Mattace Raso G., Meli R. (2019). High-Fat Diet Induces Neuroinflammation and Mitochondrial Impairment in Mice Cerebral Cortex and Synaptic Fraction. Front. Cell. Neurosci..

[46] Emerit J., Edeas M., Bricaire F. (2003). Neurodegenerative diseases and oxidative stress. Biomed. Pharmacother..

[47] Khoubnasabjafari M., Ansarin K., Jouyban A. (2015). Reliability of malondialdehyde as a biomarker of oxidative stress in psychological disorders. BioImpacts.

[48] Gumeni S., Papanagnou E.-D., Manola M.S., Trougakos I.P. (2021). Nrf2 activation induces mitophagy and reverses Parkin/Pink1 knock down-mediated neuronal and muscle degeneration phenotypes. Cell Death Dis..

[49] Butterfield D.A., Drake J., Pocernich C., Castegna A. (2001). Evidence of oxidative damage in Alzheimer’s disease brain: Central role for amyloid β-peptide. Trends Mol. Med..

[50] Yousof Ali M., Zaib S., Jannat S., Khan I. (2022). Discovery of potent and selective dual cholinesterases and β-secretase inhibitors in pomegranate as a treatment for Alzheimer’s disease. Bioorganic Chem..

[51] Le K., Song Z., Deng J., Peng X., Zhang J., Wang L., Zhou L., Bi H., Liao Z., Feng Z. (2020). Quercetin alleviates neonatal hypoxic-ischemic brain injury by inhibiting microglia-derived oxidative stress and TLR4-mediated inflammation. Agents Actions.

[52] Benameur T., Soleti R., Porro C. (2021). The Potential Neuroprotective Role of Free and Encapsulated Quercetin Mediated by miRNA against Neurological Diseases. Nutrients.

[53] Yu X., Li Y., Mu X. (2020). Effect of quercetin on PC12 Alzheimer’s disease cell model induced by Aβ25-35 and its mechanism based on sirtuin1/Nrf2/HO-1 pathway. BioMed Res. Int..

[54] Song J., Du G., Wu H., Gao X., Yang Z., Liu B., Cui S. (2021). Protective effects of quercetin on traumatic brain injury induced inflammation and oxidative stress in cortex through activating Nrf2/HO-1 pathway. Restor. Neurol. Neurosci..

[55] Saw C.L.L., Guo Y., Yang A.Y., Paredes-Gonzalez X., Ramirez C., Pung D., Kong A.-N.T. (2014). The berry constituents quercetin, kaempferol, and pterostilbene synergistically attenuate reactive oxygen species: Involvement of the Nrf2-ARE signaling pathway. Food Chem. Toxicol..

[56] Ahmad A., Khan M.M., Hoda N., Raza S.S., Javed H., Ishrat T., Ashafaq M., Ahmad E., Safhi M.M., Islam F. (2011). Quercetin Protects Against Oxidative Stress Associated Damages in a Rat Model of Transient Focal Cerebral Ischemia and Reperfusion. Neurochem. Res..

[57] Jembrek M.J., Vlainić J., Čadež V., Šegota S. (2018). Atomic force microscopy reveals new biophysical markers for monitoring subcellular changes in oxidative injury: Neuroprotective effects of quercetin at the nanoscale. PLoS ONE.

[58] Roshanzamir F., Yazdanparast R. (2014). Quercetin attenuates cell apoptosis of oxidant-stressed SK-N-MC cells while suppressing up-regulation of the defensive element, HIF-1α. Neuroscience.

[59] Tanigawa S., Fujii M., Hou D.-X. (2008). Stabilization of p53 Is Involved in Quercetin-Induced Cell Cycle Arrest and Apoptosis in HepG2 Cells. Biosci. Biotechnol. Biochem..

[60] Agarwal M.L., Taylor W.R., Chernov M.V., Chernova O.B., Stark G.R. (1998). The p53 Network. J. Biol. Chem..

[61] Xue W., Zender L., Miething C., Dickins R.A., Hernando E., Krizhanovsky V., Cordon-Cardo C., Lowe S.W. (2007). Senescence and tumour clearance is triggered by p53 restoration in murine liver carcinomas. Nature.

[62] Sablina A.A., Budanov A.V., Ilyinskaya G.V., Agapova L.S., Kravchenko J.E., Chumakov P. (2005). The antioxidant function of the p53 tumor suppressor. Nat. Med..

[63] Hafner A., Bulyk M.L., Jambhekar A., Lahav G. (2019). The multiple mechanisms that regulate p53 activity and cell fate. Nat. Rev. Mol. Cell Biol..

[64] Sharma D., Wani W., Sunkaria A., Kandimalla R., Sharma R., Verma D., Bal A., Gill K. (2016). Quercetin attenuates neuronal death against aluminum-induced neurodegeneration in the rat hippocampus. Neuroscience.

[65] Fridman J.S., Lowe S.W. (2003). Control of apoptosis by p53. Oncogene.

[66] Li X., Gu S., Ling Y., Shen C., Cao X., Xie R. (2015). p53 inhibition provides a pivotal protective effect against ischemia-reperfusion injury in vitro via mTOR signaling. Brain Res..

[67] Wolff S., Erster S., Palacios G., Moll U.M. (2008). p53’s mitochondrial translocation and MOMP action is independent of Puma and Bax and severely disrupts mitochondrial membrane integrity. Cell Res..

[68] Sun J., Wang F., Li H., Zhang H., Jin J., Chen W., Pang M., Yu J., He Y., Liu J. (2015). Neuroprotective Effect of Sodium Butyrate against Cerebral Ischemia/Reperfusion Injury in Mice. BioMed Res. Int..

[69] Maor-Nof M., Shipony Z., Lopez-Gonzalez R., Nakayama L., Zhang Y.-J., Couthouis J., Blum J.A., Castruita P.A., Linares G.R., Ruan K. (2021). p53 is a central regulator driving neurodegeneration caused by C9orf72 poly(PR). Cell.

[70] Bayazid A.B., Kim J.G., Azam S., Jeong S.A., Kim D.H., Park C.W., Lim B.O. (2021). Sodium butyrate ameliorates neurotoxicity and exerts anti-inflammatory effects in high fat diet-fed mice. Food Chem. Toxicol..

[71] Wu H., Wang Y., Zhang Y., Xu F., Chen J., Duan L., Zhang T., Wang J., Zhang F. (2020). Breaking the vicious loop between inflammation, oxidative stress and coagulation, a novel anti-thrombus insight of nattokinase by inhibiting LPS-induced inflammation and oxidative stress. Redox Biol..

[72] Xu J., Gao H., Zhang L., Rong S., Yang W., Ma C., Chen M., Huang Q., Deng Q., Huang F. (2019). Melatonin alleviates cognition impairment by antagonizing brain insulin resistance in aged rats fed a high-fat diet. J. Pineal Res..

[73] Bayazid A.B., Jeong S.A., Park C.W., Kim D.H., Lim B.O. (2022). The Anti-Inflammatory Activities of Fermented Curcuma That Contains Butyrate Mitigate DSS-Induced Colitis in Mice. Molecules.

[74] Lee B., Yeom M., Shim I., Lee H., Hahm D.-H. (2020). Protective Effects of Quercetin on Anxiety-Like Symptoms and Neuroinflammation Induced by Lipopolysaccharide in Rats. Evidence-Based Complement. Altern. Med..

[75] Wu M., Liu F., Guo Q. (2019). Quercetin attenuates hypoxia-ischemia induced brain injury in neonatal rats by inhibiting TLR4/NF-κB signaling pathway. Int. Immunopharmacol..

[76] Ashrafizadeh M., Ahmadi Z., Farkhondeh T., Samarghandian S. (2019). Autophagy as a molecular target of quercetin underlying its protective effects in human diseases. Arch. Physiol. Biochem..

[77] Han X., Xu T., Fang Q., Zhang H., Yue L., Hu G., Sun L. (2021). Quercetin hinders microglial activation to alleviate neurotoxicity via the interplay between NLRP3 inflammasome and mitophagy. Redox Biol..

[78] Crews L., Masliah E. (2010). Molecular mechanisms of neurodegeneration in Alzheimer’s disease. Hum. Mol. Genet..

[79] By S. (2013). World Alzheimer Report 2013.

[80] Wei W., Nguyen L.N., Kessels H., Hagiwara H., Sisodia S., Malinow R. (2009). Amyloid beta from axons and dendrites reduces local spine number and plasticity. Nat. Neurosci..

[81] Stoothoff W.H., Johnson G.V. (2005). Tau phosphorylation: Physiological and pathological consequences. Biochim. Biophys. Acta (BBA)-Mol. Basis Dis..

[82] Chen J., Deng X., Liu N., Li M., Liu B., Fu Q., Qu R., Ma S. (2016). Quercetin attenuates tau hyperphosphorylation and improves cognitive disorder via suppression of ER stress in a manner dependent on AMPK pathway. J. Funct. Foods.

[83] Suganthy N., Devi K.P., Nabavi S.F., Braidy N. (2016). Bioactive effects of quercetin in the central nervous system: Focusing on the mechanisms of actions. Biomed. Pharmacother..

[84] Ku T., Li B., Gao R., Zhang Y., Yan W., Ji X., Sang N. (2017). NF-κB-regulated microRNA-574-5p underlies synaptic and cognitive impairment in response to atmospheric PM2.5 aspiration. Part. Fibre Toxicol..

[85] Pang K., Jiang R., Zhang W., Yang Z., Li L.L., Shimozawa M., Lu B. (2022). An App knock-in rat model for Alzheimer’s disease exhibiting Aβ and tau pathologies, neuronal death and cognitive impairments. Cell Res..

[86] Zheng H., Koo E.H. (2006). The amyloid precursor protein: Beyond amyloid. Mol. Neurodegener..

[87] Xu M., Huang H., Mo X., Zhu Y., Chen X., Li X., Liu L. (2021). Quercetin-3-O-Glucuronide Alleviates Cognitive Deficit and Toxicity in Aβ1-42-Induced AD-Like Mice and SH-SY5Y Cells. Mol. Nutr. Food Res..

[88] Regitz C., Dußling L.M., Wenzel U. (2014). Amyloid-beta (Aβ1–42)-induced paralysis in Caenorhabditis elegans is inhibited by the polyphenol quercetin through activation of protein degradation pathways. Mol. Nutr. Food Res..

[89] Ansari M.A., Abdul H.M., Joshi G., Opii W.O., Butterfield D.A. (2009). Protective effect of quercetin in primary neurons against Aβ(1–42): Relevance to Alzheimer’s disease. J. Nutr. Biochem..

[90] Eremenko E., Mittal K., Berner O., Kamenetsky N., Nemirovsky A., Elyahu Y., Monsonego A. (2019). BDNF-producing, amyloid β-specific CD4 T cells as targeted drug-delivery vehicles in Alzheimer’s disease. EBioMedicine.

[91] Yao R.-Q., Qi D.-S., Yu H.-L., Liu J., Yang L.-H., Wu X.-X. (2012). Quercetin Attenuates Cell Apoptosis in Focal Cerebral Ischemia Rat Brain Via Activation of BDNF–TrkB–PI3K/Akt Signaling Pathway. Neurochem. Res..

[92] Arias N., Macarulla M.T., Aguirre L., Martínez-Castaño M.G., Portillo M.P. (2013). Quercetin can reduce insulin resistance without decreasing adipose tissue and skeletal muscle fat accumulation. Genes Nutr..

[93] Osama A., Zhang J., Yao J., Yao X., Fang J. (2020). Nrf2: A dark horse in Alzheimer’s disease treatment. Ageing Res. Rev..

[94] Bettens K., Sleegers K., van Broeckhoven C. (2013). Genetic insights in Alzheimer’s disease. Lancet Neurol..

[95] Association A.S. (2019). 2019 Alzheimer’s disease facts and figures. Alzheimer’s Dement..

[96] Liu L., MacKenzie K.R., Putluri N., Maletić-Savatić M., Bellen H.J. (2017). The Glia-Neuron Lactate Shuttle and Elevated ROS Promote Lipid Synthesis in Neurons and Lipid Droplet Accumulation in Glia via APOE/D. Cell Metab..

[97] Boesch-Saadatmandi C., Wolffram S., Minihane A.M., Rimbach G. (2008). Effect of apoE genotype and dietary quercetin on blood lipids and TNF-α levels in apoE3 and apoE4 targeted gene replacement mice. Br. J. Nutr..

[98] Plump A.S., Breslow J.L. (1995). Apolipoprotein E and the apolipoprotein E-deficient mouse. Annu. Rev. Nutr..

[99] Liu C.-C., Kanekiyo T., Xu H., Bu G. (2013). Apolipoprotein E and Alzheimer disease: Risk, mechanisms and therapy. Nat. Rev. Neurol..

[100] Rhinn H., Fujita R., Qiang L., Cheng R., Lee J.H., Abeliovich A. (2013). Integrative genomics identifies APOE ε4 effectors in Alzheimer’s disease. Nature.

[101] Bellenguez C., Küçükali F., Jansen I.E., Kleineidam L., Moreno-Grau S., Amin N., Naj A.C., Campos-Martin R., Grenier-Boley B., Andrade V. (2022). New insights into the genetic etiology of Alzheimer’s disease and related dementias. Nat. Genet..

[102] Tachibana M., Holm M.-L., Liu C.-C., Shinohara M., Aikawa T., Oue H., Yamazaki Y., Martens Y.A., Murray M.E., Sullivan P.M. (2019). APOE4-mediated amyloid-β pathology depends on its neuronal receptor LRP1. J. Clin. Investig..

[103] Carmona S., Zahs K., Wu E., Dakin K., Bras J., Guerreiro R. (2018). The role of TREM2 in Alzheimer’s disease and other neurodegenerative disorders. Lancet Neurol..

[104] Lee J.-H., Yang D.S., Goulbourne C.N., Im E., Stavrides P., Pensalfini A., Nixon R.A. (2022). Faulty autolysosome acidification in Alzheimer’s disease mouse models induces autophagic build-up of Aβ in neurons, yielding senile plaques. Nat. Neurosci..

[105] Wadhwani A.R., Affaneh A., Van Gulden S., Kessler J.A. (2019). Neuronal apolipoprotein E4 increases cell death and phosphorylated tau release in alzheimer disease. Ann. Neurol..

[106] Zheng X.J., Chen W.L., Yi J., Li W., Liu J.Y., Fu W.Q., Wang J.H. (2022). Apolipoprotein C1 promotes glioblastoma tumorigenesis by reducing KEAP1/NRF2 and CBS-regulated ferroptosis. Acta Pharmacol. Sin..

[107] He K., Nie L., Zhou Q., Rahman S.U., Liu J., Yang X., Li S. (2019). Proteomic Profiles of the Early Mitochondrial Changes in APP/PS1 and ApoE4 Transgenic Mice Models of Alzheimer’s Disease. J. Proteome Res..

[108] Zhu M., Han S., Fink A.L. (2013). Oxidized quercetin inhibits α-synuclein fibrillization. Biochim. Biophys. Acta (BBA)-Gen. Subj..

[109] Stefanis L. (2012). α-Synuclein in Parkinson’s disease. Cold Spring Harb. Perspect. Med..

[110] Wang W.-W., Han R., He H.J., Li J., Chen S.Y., Gu Y., Xie C. (2021). Administration of quercetin improves mitochondria quality control and protects the neurons in 6-OHDA-lesioned Parkinson’s disease models. Aging.

[111] Das N., Sharma S. (2015). Peroxisome Proliferator Activated Receptor Gamma Coactivator 1 Alpha: An Emerging Target for Neuroprotection in Parkinson’s Disease. CNS Neurol. Disord-Drug Targets.

[112] Xiao N., Mei F., Sun Y., Pan G., Liu B., Liu K. (2014). Quercetin, Luteolin, and Epigallocatechin Gallate Promote Glucose Disposal in Adipocytes with Regulation of AMP-Activated Kinase and/or Sirtuin 1 Activity. Planta Medica.

[113] Gonzales M.M., Garbarino V.R., Marques Zilli E., Petersen R.C., Kirkland J.L., Tchkonia T., Orr M.E. (2022). Senolytic Therapy to Modulate the Progression of Alzheimer’s Disease (SToMP-AD): A Pilot Clinical Trial. J. Prev. Alzheimer’s Dis..

[114] Nishihira J., Nishimura M., Kurimoto M., Kagami-Katsuyama H., Hattori H., Nakagawa T., Muro T., Kobori M. (2021). The effect of 24-week continuous intake of quercetin-rich onion on age-related cognitive decline in healthy elderly people: A randomized, double-blind, placebo-controlled, parallel-group comparative clinical trial. J. Clin. Biochem. Nutr..

[115] Rinwa P., Kumar A. (2013). Quercetin along with piperine prevents cognitive dysfunction, oxidative stress and neuro-inflammation associated with mouse model of chronic unpredictable stress. Arch. Pharmacal Res..

[116] Amanzadeh E., Esmaeili A., Rahgozar S., Nourbakhshnia M. (2019). Application of quercetin in neurological disorders: From nutrition to nanomedicine. Rev. Neurosci..

[117] Collaboration B.P.L.T.T. (2003). Effects of different blood-pressure-lowering regimens on major cardiovascular events: Results of prospectively-designed overviews of randomised trials. Lancet.

[118] Harwood M., Danielewska-Nikiel B., Borzelleca J.F., Flamm G.W., Williams G.M., Lines T.C. (2007). A critical review of the data related to the safety of quercetin and lack of evidence of in vivo toxicity, including lack of genotoxic/carcinogenic properties. Food Chem. Toxicol..

